# Airway Remodeling Factors During Early-Life Rhinovirus Infection and the Effect of Premature Birth

**DOI:** 10.3389/fped.2021.610478

**Published:** 2021-02-26

**Authors:** Xilei XuChen, Jered Weinstock, Maria Arroyo, Kyle Salka, Elizabeth Chorvinsky, Karima Abutaleb, Hector Aguilar, Ryan Kahanowitch, Carlos E. Rodríguez-Martínez, Geovanny F. Perez, Maria J. Gutierrez, Gustavo Nino

**Affiliations:** ^1^Division of Pediatric Pulmonary and Sleep Medicine, Children's National Hospital, George Washington University, Washington, DC, United States; ^2^Department of Pediatrics, School of Medicine, Universidad Nacional de Colombia, Bogota, Colombia; ^3^Department of Pediatric Pulmonology and Pediatric Critical Care Medicine, School of Medicine, Universidad El Bosque, Bogota, Colombia; ^4^Division of Pediatric Pulmonology, Oishei Children's Hospital, University at Buffalo, Buffalo, NY, United States; ^5^Division of Pediatric Allergy and Immunology, Johns Hopkins University, Baltimore, MD, United States

**Keywords:** rhinovirus (RV), airway remodeling, prematurity, infancy, growth factor

## Abstract

**Background:** Early rhinovirus (RV) infection is a strong risk factor for asthma development. Airway remodeling factors play a key role in the progression of the asthmatic condition. We hypothesized that RV infection in young children elicits the secretion of growth factors implicated in airway remodeling and asthma progression.

**Methods:** We examined the nasal airway production of remodeling factors in children ( ≤ 2 years old) hospitalized due to PCR-confirmed RV infection. Airway remodeling proteins included: MMP-1, MMP-2, MMP-7, MMP-9, MMP-10, TIMP-1, TIMP-2, EGF, Angiopoietin-2, G-CSF, BMP-9, Endoglin, Endothelin-1, Leptin, FGF-1, Follistatin, HGF, HB-EGF, PLGF, VEGF-A, VEGF-C, VEGF-D, FGF-2, TGF-β1, TGF-β2, TGF-β3, PDGF AA, PDGF BB, SPARC, Periostin, OPN, and TGF-α.

**Results:** A total of 43 young children comprising RV cases (*n* = 26) and uninfected controls (*n* = 17) were included. Early RV infection was linked to (1) enhanced production of several remodeling factors (e.g., HGF, TGFα), (2) lower MMP-9/TIMP-2 and MMP-2/TIMP-2 ratios, and (3) increased MMP-10/TIMP-1 ratios. We also found that relative to term infants, severely premature children had reduced MMP-9/TIMP-2 ratios at baseline.

**Conclusion:** RV infection in young children elicits the airway secretion of growth factors implicated in angiogenesis, fibrosis, and extracellular matrix deposition. Our results highlight the potential of investigating virus-induced airway remodeling growth factors during early infancy to monitor and potentially prevent chronic progression of respiratory disorders in all ages.

## Introduction

Human rhinovirus (RV) has been identified as the most common cause of wheezing illnesses and asthma exacerbations in all ages (1). Moreover, RV-induced wheezing illnesses during the first 2–3 years of life are considered the strongest risk factor (10 times increased odds) for the development of asthma beyond childhood (2). This RV-related risk is even higher in individuals with genetic predisposition (e.g., variants at the 17q21 locus) (3) and in those with early life aeroallergen sensitization (4). While most research has focused on how RV contributes to the initiation and progression of allergic inflammation (4), there is evidence that RV may directly induce remodeling factors that promote chronic changes in the airways (5). Airway remodeling is a feature of several chronic airway diseases like asthma and encompasses permanent structural changes including sub-epithelial thickening, extracellular matrix deposition or fibrosis, and an increase in airway smooth muscle mass (6–8). These airway remodeling changes have been described in young children with airflow limitation even before any diagnosis of asthma (9, 10). Nonetheless, whether RV infections induce the production of remodeling factors in the airways of young children remains to be determined.

Prior studies have shown that RV can upregulate *in-vitro* remodeling factors in airway epithelial cells including vascular endothelial growth factor (VEGF), fibroblast growth factor (FGF), and specific matrix metalloproteinases (MMP) (11–14). However, the specific remodeling factors produced in the airways of young children with naturally occurring RV infections are presently unknown. Filling this gap can be critically important as it may lead to the identification of novel targetable molecular pathways that mediate chronic progression of RV-induced wheezing illnesses during childhood. This new knowledge would be particularly impactful for children born severely premature as they are highly susceptible to develop severe and recurrent RV infections as well as other virus-induced wheezing illnesses during infancy and beyond childhood (15–20).

The goal of this study was to define for the first time the specific airway remodeling signatures induced by RV during early life. For this purpose, we determined protein levels of 32 remodeling factors in the nasal secretions of young children (≤ 2 years old) hospitalized with severe RV infections and in uninfected controls. Our central hypothesis was that RV infection in young children elicits the airway secretion of growth factors implicated in angiogenesis, fibrosis, and extracellular matrix deposition. Additional analyses were conducted to identify the effect of severe prematurity in the RV-induced airway remodeling signatures in young children.

## Methods

### Study Subjects

Nasal airway secretions were collected in patients ≤ 2 years old of age with PCR-confirmed RV infection. All subjects were enrolled during the hospital admission for RV infection. We included age-matched controls without viral respiratory infection (negative viral PCR) recruited during non-respiratory hospitalizations or outpatient/emergency department visits. Baseline characteristics were obtained by reviewing electronic medical records (EMR) and are presented in Table 1. Our sample was obtained while they were undergoing diagnostic nasal lavage (respiratory virus detection by PCR) at Children's National Hospital. RV positive (RV-infected group) or negative virus status (control group) was confirmed by a viral multiplex PCR panel for 12 targets (RV, RSV A, RSV B, HMPV, parainfluenza 1–3, influenza A and B, H1N1, H1N3, Adenovirus) used for clinical purposes (Luminex, TX, USA). Mixed viral infections were not included in the RV positive group. For the purpose of the study, severe prematurity was defined by a gestational age (GA) of ≤ 32 weeks. The study protocol (Pro00003441) was approved by the Institutional Review Board (IRB) of Children's National Hospital, Washington D.C.

**Table 1:** Baseline characteristics of the study subjects.

	**Term**		**Severe premature**	
	**Control**	**RV**	**Control**	**RV**
*N* (%)	6 (14)	9 (21)	11 (26)	17 (39)
Age (years), mean (SD)	0.48 (0.46)	1.22 (0.68)	0.66 (0.43)	1.10 (0.60)
GA (weeks) mean (SD)	38.5 (1.05)	37 (1.93)	25.8 (1.94)	25.6 (2.2)
Male gender, n (%)	4 (66)	3 (33)	10 (90)	11 (65)
Black/African American, n (%)	2 (33)	4 (44)	7 (64)	11 (65)

*RV, Rhinovirus; GA, Gestational Age; SD, Standard Deviation*.

### Nasal Washing Collection and Cytokine Measurements

We used a standard nasal lavage technique consisting of gently washing the nasal cavity with 3–4 mL sterile normal saline as previously described (19). All samples were obtained with the same protocol to minimize variable recovery during nasal aspirate collection. Nasal washings were analyzed for protein levels of 32 growth factors implicated in remodeling and angiogenesis using a commercially available multiplex magnetic bead immunoassay (Millipore, MA, USA) according to the manufacturers' instructions and with provided standards and quality controls. Protein levels below the immunoassay's limit of detection (LOD) were assigned the LOD provided in the assay. The following analytes were included: matrix metalloproteinases (MMP-1, MMP-2, MMP-7, MMP-9, and MMP-10), Tissue inhibitor of metalloproteinases (TIMP-1 and TIMP-2), Epidermal growth factor (EGF), Angiopoietin-2, Granulocyte colony-stimulating factor (G-CSF), Bone morphogenic protein-9 (BMP-9), Endoglin, Endothelin-1, Leptin, Fibroblast growth factors (FGF-1 and FGF-2), Follistatin, Hepatic growth factor (HGF), Heparin-binding EGF-like growth factor (HB-EGF), Placental growth factor (PLGF), vascular endothelial growth factors (VEGF-A, VEGF-C, VEGF-D), transforming growth factors (TGF-α, TGF-β1, TGF-β2, and TGF-β3), Platelet-derived growth factors (PDGF-AA and PDGF-BB), Secreted protein acidic and rich in cysteine (SPARC), Periostin and Osteopontin (OPN).

### Statistical Analysis

Differences between groups on continuous variables were analyzed using the non-parametric Mann-Whitney *U* test. Benjamini–Hochberg false discovery rate (FDR) method was used to adjust for multiple comparisons. Associations between categorical variables were analyzed using the X^2^ test. RV-induced changes in MMP/TIMP ratios were adjusted by pertinent covariates using generalized linear regression models. All statistical tests were two-tailed, and the significance level used was *p* < 0.05. The data were analyzed with the Minitab Statistical Package V.19.1. (Minitab, Inc., State College, PA).

## Results

### Baseline Characteristics

Forty-three children aged ≤ 2 years of age were included in this study. The total study sample comprised young children hospitalized due to PCR-confirmed RV infections (*n* = 26) and age-matched uninfected controls (*n* = 17). To examine the effect of severe prematurity in our main outcome (secretion of airway remodeling factors) we included RV infected and control individuals born full-term or severely premature (≤ 32 weeks GA). Baseline characteristics of these groups are shown in Table 1.

### Airway Remodeling Factors in Young Children During RV Infection

Analysis of individual nasal protein levels of different airway remodeling factors demonstrated that young children hospitalized due to severe RV infection had a significant increase in the production of several soluble growth factors involved in remodeling as compared to uninfected controls. Table 2 shows the complete list of airway remodeling factors according to RV status. We found that during RV infection young children had higher nasal airway responses of TIMP-2, HGF, TGF-α, and MMP-10 (Table 2). Other remodeling factors such as Endoglin, G-CSF, VEGF-D, Leptin, MMP-9, FGF-2, and HB-EGF trended to be increased during RV infection but were not significant after FDR adjustment (Table 2).

**Table 2:** Comparison of airway remodeling factors in young children hospitalized with rhinovirus infection and uninfected controls.

**Nasal airway protein levels (pg/ml)**	**Control (*n =* 17)**	**Rhinovirus (*n =* 26)**	***P-*value**	Adj. *P-*value^*^
TIMP-2	991.16	8996.5	0.0001	**0.003**
HGF	50.74	806.82	0.001	**0.017**
TGFα	0.81	21.71	0.001	**0.011**
MMP-10	862.51	4,583	0.004	**0.033**
Endoglin	15.69	26.84	0.012	0.079
G-CSF	51.88	165.62	0.015	0.082
VEGF-D	5.96	4.48	0.016	0.075
Leptin	86.96	74.05	0.02	0.083
MMP-9	6542	13,289	0.025	0.092
FGF-2	17.78	22.78	0.031	0.102
HB-EGF	4.25	14.9	0.047	0.141
VEGF-A	306.7	736.82	0.063	0.160
FGF-1	6.10	8.11	0.104	0.245
MMP-2	216.03	316.19	0.14	0.272
MMP-7	787.77	371.11	0.16	0.293
MMP-1	21.67	11.91	0.48	0.587
TIMP-1	10,042	10787.5	0.518	0.611
EGF	47.32	100.5	0.465	0.590
Angiopoietin-2	8.54	6.31	0.058	0.160
BMP-9	0.95	1.05	0.356	0.490
Endothelin-1	4.49	3.91	0.228	0.376
Follistatin	29.74	24.31	0.214	0.372
PLGF	1.95	6.11	0.249	0.391
VEGF-C	19.26	54.94	0.127	0.262
TGFβ1	10942.5	6,835	0.53	0.603
TGFβ2	15247.5	14,299	0.339	0.486
TGFβ3	2,751	2,622	0.83	0.856
PDGF AA	950.84	913.41	0.786	0.837
PDGF BB	6,673	6,673	0.648	0.713
SPARC	0.25	0.25	0.907	0.907
Periostin	0.14	0.27	0.386	0.510
OPN	327.32	267.14	0.304	0.456

* *p value adjusted by false discovery rate (FDR). Bold values denote statistical significance (p < 0.05)*.

To examine the effect of severe prematurity in the production of airway remodeling factors during early life, we first examined the baseline airway production of these molecules in uninfected young children. We found that severely premature children trended to have increased baseline production of several remodeling growth factors (e.g., MMP-2 and TGF-α) but there were no significant differences relative to term infants after FDR adjustment (Table 3). We also contrasted airway remodeling factors in young children born full-term or severely premature during RV infections. We found that relative to full-term children, individuals born severely premature had overall similar RV-induced production of remodeling factors (Table 3). Collectively, these results suggest that RV infection in term and premature young children elicits the airway secretion of specific growth factors implicated in angiogenesis, fibrosis, and extracellular matrix deposition.

**Table 3:** Comparison of airway remodeling factors at baseline and during RV infection according to severe prematurity status.

	**Baseline levels**				**RV-induced levels**			
**Nasal airway protein levels (pg/ml)**	**Term**	**Premature**	***P-*value**	Adj. *P-*value^*^	**Term**	**Premature**	***P-*value**	Adj. *P-*value^*^
TIMP-2	487.48	1,314	0.11	0.196	9,090	8,580	0.89	0.949
HGF	34.81	144.17	0.079	0.194	899.34	806.82	0.95	0.981
TGFα	1.93	14.75	0.021	0.168	21.22	16.64	0.75	1.000
MMP-10	332.96	1,437	0.11	0.220	5888.5	3,106	0.29	1.000
Endoglin	14.67	19.63	0.11	0.207	17.02	32.98	0.048	0.768
G-CSF	25.97	62.48	0.37	0.538	451.03	124.41	0.2	1.000
VEGF-D	4.59	6.9	0.018	0.192	4.48	4.48	0.6	0.873
Leptin	76.63	98.56	0.024	0.154	70.17	74.05	0.4	0.914
MMP-9	6,386	6,542	0.62	0.763	15709.5	12,622	0.48	0.960
FGF-2	13.7	19	0.039	0.178	23.43	20.87	0.56	0.943
HB-EGF	2.34	6.17	0.044	0.176	14	15.75	0.77	0.986
VEGF-A	178.7	429.84	0.035	0.187	761.66	736.82	0.6	0.914
FGF-1	4.75	7.35	0.37	0.564	5.51	9.19	0.048	1.000
MMP-2	97.54	322.8	0.009	0.144	398.68	335.46	0.2	1.000
MMP-7	418.02	1,737	0.07	0.187	276.08	442.47	0.77	0.948
MMP-1	16.56	21.67	1.0	1.000	13.09	11.91	0.45	0.960
TIMP-1	7,623	10,042	0.37	0.493	16,271	9,941	0.57	0.912
EGF	39.66	67.8	0.088	0.201	140.57	100.5	0.73	1.000
Angiopoietin-2	6.61	8.84	0.063	0.202	5.04	6.61	0.086	0.917
BMP-9	0.775	1.1	0.007	0.224	0.97	1.05	0.38	0.935
Endothelin-1	3.49	5.62	0.13	0.219	3.78	4.34	0.34	0.989
Follistatin	22.58	30.64	0.37	0.515	26.55	21.91	0.79	0.936
PLGF	1.33	3.88	0.044	0.156	5.65	7.42	1	1.000
VEGF-C	8.93	21.3	0.063	0.183	41.76	54.94	0.88	0.971
TGFβ1	15,050	6,835	0.49	0.627	6,835	8417.5	0.85	0.971
TGFβ2	13,197	15,580	0.73	0.865	13,197	14,299	0.55	0.978
TGFβ3	1,774	2,815	0.78	0.861	1,806	3921.5	0.24	0.960
PDGF AA	1,034	867.69	0.95	0.981	913.41	909.71	0.53	0.998
PDGF BB	8136	6,673	0.78	0.832	10,000	4215.5	0.33	1.000
SPARC	0.33	0.19	0.31	0.496	0.22	0.25	0.24	1.000
Periostin	0.095	0.26	0.088	0.188	0.35	0.11	0.35	0.933
OPN	268.65	337.84	0.76	0.869	241.18	288	0.228	1.000

* *p-value adjusted by false discovery rate (FDR)*.

### Matrix Metalloproteinase/Tissue Inhibitor Metalloproteinase Ratios in Young Children During RV Infection

**Figure 1:**
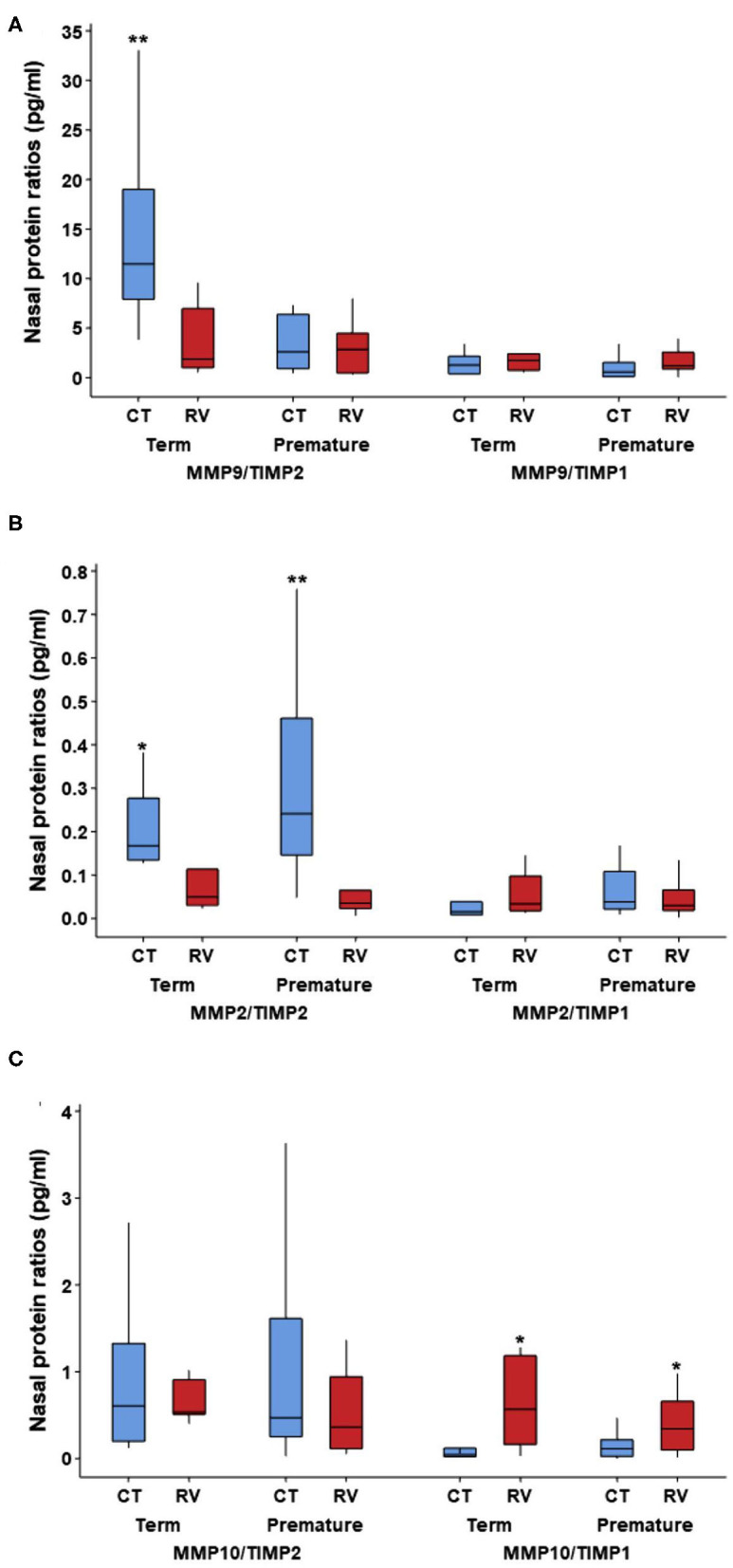
Nasal airway MMP/TIMP ratios at baseline and during RV infection in young children according to severe prematurity status. Comparison of nasal protein levels of matrix metalloproteinases (MMP) and tissue inhibitor metalloproteinases (TIMP) ratios in non-infected controls (CT) and young children with rhinovirus (RV) infection. **(A)** MMP9/TIMP2 is increased in CT term relative to RV infection and CT in premature; **(B)** MMP2/TIMP2 is increased in CT relative to RV in term and premature; **(C)** MMP10/TIMP1 is increased in RV infection relative to CT in term and premature. Symbols represent ^*^p < 0.05 and ^**^p < 0.01; no other pairwise comparisons were significant. Data presented as boxplots representing 25th−75th percentiles.

Since our individual molecular analyses identified that RV elicits the production of different matrix metalloproteinase (MMP) and tissue inhibitor metalloproteinases (TIMP) in the airways of young children, we next examined MMP/TIMP ratios across our study groups. We focused on specific MMPs (MMP-9, MMP-2, MMP-10) relative to TIMP-1 and TIMP-2 levels, as the ratios of these enzymes have been implicated in the regulation of airway remodeling (21–26) and their individual levels showed significant differences in our study (Table 2). Our results demonstrated that MMP-9/TIMP-2 ratios were significantly decreased in term children after RV infection (Figure 1A). MMP-9/TIMP-2 ratios were low at baseline in severely premature subjects and thus were not further decreased during RV infection (Figure 1A). We also identified that RV infection in young children was linked to a decreased MMP-2/TIMP-2 and an increased MMP-10/TIMP-1 ratios. The later RV-induced changes were similar in term and premature children (Figures 1B,C). Multivariate analyses demonstrated that the RV-induced changes in the MMP-9/TIMP-2, MMP-2/TIMP-2, and MMP10/TIMP-1 ratios were independent of age, sex, and race/ethnicity (Table 4). Taken together, these data demonstrate that RV infection elicits significant changes in the enzymatic MMP/TIMP balance in the airways of young children.

**Table 4:** Multivariate analysis of RV-induced changes in nasal airway MMP/TIMP ratios in young children.

**Predictors**	**MMP-9/TIMP-2 levels**		**MMP-2/TIMP-2 levels**		**MMP10/TIMP-1 levels**	
	**Effect estimate (β)**	***P-*value**	**Effect estimate (β)**	***P-*value**	**Effect estimate (β)**	***P-*value**
Rhinovirus	−4.8	**0.031**	−0.2141	**0.002**	0.536	**0.037**
Age	−1	0.583	−0.034	0.541	−0.163	0.439
Sex^*^	−4.05	0.075	−0.0179	0.793	−0.367	0.158
Race/ethnicity^**^	−3.19	0.119	−0.0022	0.971	−0.17	0.466

* *Male reference group*,

** *Black/African American reference group. Bold values denote statistical significance (p < 0.05)*.

## Discussion

The goal of this study was to define for the first time the specific airway remodeling signatures induced by RV during early life. This is a high-impact Research Topic because RV is the most common cause of wheezing illnesses and early life RV infections confer a significantly increased risk for the development of asthma beyond childhood (2). Our results demonstrate that RV infection in young children elicits the airway secretion of growth factors implicated in angiogenesis, fibrosis, and extracellular matrix deposition. We also found intrinsic differences in the baseline airway remodeling factors between young children born full-term and those born severely premature. Our results highlight the potential of investigating virus-induced airway remodeling growth factors during early infancy to monitor and potentially prevent chronic respiratory disorders in all ages.

There is prior evidence that RV can induce growth factors implicated in healing and remodeling in airway epithelial cells (11–14). In this study, we found that the overall top growth factors induced by RV in young children were transforming growth factor alpha (TGF-α) and hepatic growth factor (HGF) (Table 2). TGF-α binds the epidermal growth factor receptor (EGFR) in the epithelium to exert a myriad of potent effects in the airways including maturation, remodeling and increased mucous production (27). HGF is produced by mesenchymal cells and after binding its receptor in the airway epithelium (c-Met), it induces many protective effects including inhibition of apoptosis and oxidative stress injuries (28) as well as attenuation of airway hyperresponsiveness, inflammation, and remodeling (29). The production of TGF-α and HGF has been described during RV infection in cellular models (30, 31), but to the best of our knowledge, our study is the first to report this in young children during naturally occurring RV infections. It is also important to emphasize that several other remodeling growth factors were increased during RV infections in term and premature infants (Tables 2, 3). These growth factors and other related molecules are likely to collectively form a network of signaling responses to orchestrate healing, repair as well as fibrosis in response to RV in early life. As a result, their collective function, rather than individual effects, is likely to play a significant role not only in acute RV infections but also during subsequent airway responses after recovery.

Along with the effect of RV in multiple airway growth factors, we also found that this virus altered the balance of matrix metalloproteinase (MMP) and tissue inhibitor metalloproteinases (TIMP) enzymes in the airways of young children. This is relevant because the imbalance between MMP/TIMP molecules is considered a major theory to explain the progression of airway remodeling (21–26). MMPs, and their putative inhibitors TIMPs, are the key enzymes responsible for extracellular matrix degradation and thus regulate healing and repair processes as well as the development of fibrotic changes (21–26). The ratios of these molecules have been directly correlated with airway remodeling changes in humans (21–26). Low sputum MMP-9/TIMP2 ratios are associated with airway narrowing in smokers with asthma (22) and MMP-9/TIMP-1 ratios correlate inversely with airway wall thickness assessed by CT scanning in asthmatics (23). In our study, we found that RV infection is associated with reduced MMP-9/TIMP2 ratios suggesting that this virus may play a role inducing remodeling changes in early life. Interestingly, severely premature children had low MMP-9/TIMP2 levels at baseline, which might indicate the presence of chronic remodeling changes due to prematurity-related injuries and/or developmental deficits. We also found that RV decreases MMP2/TIMP-2 levels while increasing MMP-10/TIMP-1 ratios. This profile also supports that RV promotes airway remodeling changes in young children. MMP-2 is generally considered protective for the airways (24), however MMP-10 is induced by viral infections (25) and is linked to increased submucosal thickness (26). In summary, our study clearly demonstrated that RV induces an imbalance between MMP/TIMP molecules. However, it is important to emphasize that in early life these changes could be part of the normal airway development and/or repairing responses after virus-induced injuries. Additional studies are needed to understand if RV-induced MMP/TIMP imbalances have any deleterious effects in the progression of respiratory illnesses in young children born at term or severely premature.

In addition to reduced MMP-9/TIMP2 ratios, we have previously shown that severely premature children have increased airway secretion of TH2 and TH17 cytokines during RV infections (19). Our current findings complement this initial observation demonstrating that RV not only elicits an airway pro-inflammatory state but heightens the production of angiogenic and pro-fibrotic factors in severely premature infants. While some RV-induced airway remodeling factors are likely to be protective, some may contribute to increased respiratory morbidity. Defining the clinical relevance of these changes will be important as RV infections are a major cause of health care utilization, hospitalization, and morbidity in premature children (15–20). The later may relate to deficits in lung function such as higher resistance of the respiratory system during early infancy (32, 33). Since airway remodeling contributes to changes in the bronchial wall leading to increased resistance (6–8), it is possible that RV-induced angiogenic and pro-fibrotic factors may be clinically relevant by predisposing infants to severe and recurrent virus-induced wheezing illnesses in early childhood, particularly in those born prematurely. This important possibility needs to be considered in future larger longitudinal studies.

Our study has a number of strengths and some limitations. We were able to investigate multiple remodeling growth factors not previously reported in the literature of RV infection in young children. In that sense, our study successfully provided important novel molecular insights on the potential effects of viruses in the regulation of remodeling responses during early childhood. The main limitations of the present study are the sample size and the cross-sectional nature of the sampling. It is important to emphasize that this is a small pilot study and our findings need prospective validation and clinical correlation. Indeed, prospective sampling is needed to confirm the increase in remodeling factors once RV infection occurs in early life, and whether this normalizes later or continues well into late childhood. In addition, the study was conducted in a specialized, tertiary referral hospital, which makes it likely that the patients included represent the extreme of the spectrum of severity of infants born premature, thus limiting the generalization of results to other contexts. Another important caveat is that the concentrations of growth factors in the nasal passages do not necessarily reflect those in the lower airway. Our study included only nasal samples as bronchial specimens cannot be readily obtained during acute RV infections in young children. However, in this regard, there is a large body of literature demonstrating nasal and bronchial respiratory epithelial similarities with approximately 91% homology in the expressed genes between the two sites (34). Accordingly, although further studies including bronchial specimens are still needed, nasal sampling is a non-invasive approach to examining virus-induced airway responses in infants and young children (35–37). Because the focus of the study was the quantification of remodeling factors we did not include viral loads, cytokines or clinical data (e.g., nasal lavage cell counts, severity of infection/duration of hospitalization), which are important variables during RV infections in infants. Finally, because EMR information did not provide a complete medical history in all cases, we did not include in the analyses some potentially important factors for RV infection severity such as eczema, socioeconomic status and environmental factors (e.g., smoking and daycare attendance), prematurity-related factors (O_2_, chronic lung disease, steroids use) and as is the case for other observational epidemiologic studies, residual confounding cannot be excluded, so interpretation of our results needs to be cautious.

In conclusion, our study identified that (1) RV infection in term and premature young children is linked to an enhanced secretion of growth factors implicated in airway remodeling (TIMP-2, HGF, TGF-α, and MMP-10); and (2) severely premature children have reduced nasal airway MMP-9/TIMP-2 ratios at baseline. Further investigating the underlying molecular mechanisms linking early RV infection to airway remodeling could lead to novel biomarkers and targeted interventions to prevent chronic progression of respiratory diseases in children.

## Data Availability Statement

The original contributions presented in the study are included in the article/supplementary material, further inquiries can be directed to the corresponding author/s.

## Ethics Statement

The studies involving human participants were reviewed and approved by Institutional Review Board (IRB) of Children's National Hospital, Washington D.C. Written informed consent to participate in this study was provided by the participants' legal guardian/next of kin.

## Author Contributions

XX-C and GN: study design. XX-C, JW, MA, HA, RK, GP, and GN: data collection. GN, CR-M, JW, MG, and XX-C: analysis. All authors: manuscript drafting, editing, approval.

## Conflict of Interest

The authors declare that the research was conducted in the absence of any commercial or financial relationships that could be construed as a potential conflict of interest.
